# Synchronization to a bouncing ball with a realistic motion trajectory

**DOI:** 10.1038/srep11974

**Published:** 2015-07-07

**Authors:** Lingyu Gan, Yingyu Huang, Liang Zhou, Cheng Qian, Xiang Wu

**Affiliations:** 1 Department of Psychology, Sun Yat-Sen University, Building 313, 135 Xingang west road, Guangzhou, Guangdong, China, 510275.

## Abstract

Daily music experience involves synchronizing movements in time with a perceived periodic beat. It has been established for over a century that beat synchronization is less stable for the visual than for the auditory modality. This auditory advantage of beat synchronization gives rise to the hypotheses that the neural and evolutionary mechanisms underlying beat synchronization are modality-specific. Here, however, we found that synchronization to a periodically bouncing ball with a realistic motion trajectory was not less stable than synchronization to an auditory metronome. This finding challenges the auditory advantage of beat synchronization, and has important implications for the understanding of the biological substrates of beat synchronization.

Most forms of music have a perceived periodic beat (or pulse), and people often move (e.g., tap a finger or foot) in synchrony with the beat^1^. The capacity to entrain motor behaviors to a beat is predictive (i.e., on average, taps slightly precede event onsets when tapping to a beat) and flexible (i.e., synchronization to an auditory beat is accurate for inter-beat intervals ranging from 300 to 900 ms, with the most preferred inter-beat intervals being approximately 600 ms)^2^. One key feature of beat synchronization is the auditory advantage that has been established for over a century; synchronization is less stable to a visual (e.g., flashes of a light) than to an auditory beat (e.g., an auditory metronome)^3^. This modality bias of beat synchronization has essential impacts on the understanding of the biological substrates of sensorimotor integration and human evolution. Tighter connections between the auditory and motor cortices than between the visual and motor cortices have been suggested for beat synchronization^4^,^5^,^6^. Human-like beat synchronization behaviors have primarily been observed in animals with vocal learning ability (e.g., parrots). It has thus been suggested that vocal learning drives the evolution of flexible beat synchronization and that flexible beat synchronization is a unique brain function that is shared by humans and only a few other species^6^,^7^.

However, recent advances have shown that synchronization to a visual beat can be improved using moving stimuli instead of a conventional flashing light ^for review, see^ 8. Specifically, Iversen *et al.*^9^ reported that tapping stability to a periodically bouncing ball, which had a velocity that varied according to a rectified sinusoid, was close to that to an auditory metronome. Hove *et al.*^10^ later found an advantage for an auditory metronome over the bouncing ball. People usually move along with moving visual stimuli that contain spatiotemporal information rather than with a visual stimulus without spatial changes, such as a flashing light that lacks ecological validity^11^. Therefore, these findings indicate that synchronization to a visual beat composed of realistically moving stimuli is almost as good as synchronization to an auditory metronome, although a slight auditory advantage still persisted in studies employing moving visual stimuli.

Since synchronization to a visual beat can be improved by employing realistically moving stimuli, its performance may be further improved and could reach the level of synchronization to an auditory beat if the moving stimuli could be more realistic. The present study investigated this hypothesis. Previous studies have shown that periodically bouncing ball stimuli moving with a rectified sinusoidal velocity profile are the most effective moving stimuli in improving synchronization to a visual beat^9^,^10^. We designed a bouncing ball that was even more realistic by two manipulations. (1) The velocity of the current bouncing ball was varied by simulating the effect of gravity because people can precisely interact with free-falling objects^12^. (2) Movement smoothness was carefully controlled to avoid movement discontinuities that could potentially impact the subjects’ judgment of the trajectory. Synchronization to an isochronous rhythmic sequence that was composed of an auditory tone, a visual flash, or the current bouncing ball was examined in experiment 1 using a 600 or 900 ms inter-onset interval (IOI) (i.e., inter-beat interval). In experiment 2, the auditory tone sequence and the current bouncing ball sequence were compared for more IOIs (300, 500, 700, and 900 ms). After that, the current bouncing ball sequence was compared with a bouncing ball sequence with a sinusoidally varying velocity in control experiment 1, and the effect of each of the two manipulations of the current bouncing ball sequence was studied in control experiment 2. Moreover, the current bouncing ball sequence was rotated 90° counterclockwise and was compared with the non-rotated sequence in control experiment 3. A 600 ms IOI was used in all control experiments.

## Results

**Figure 1:**
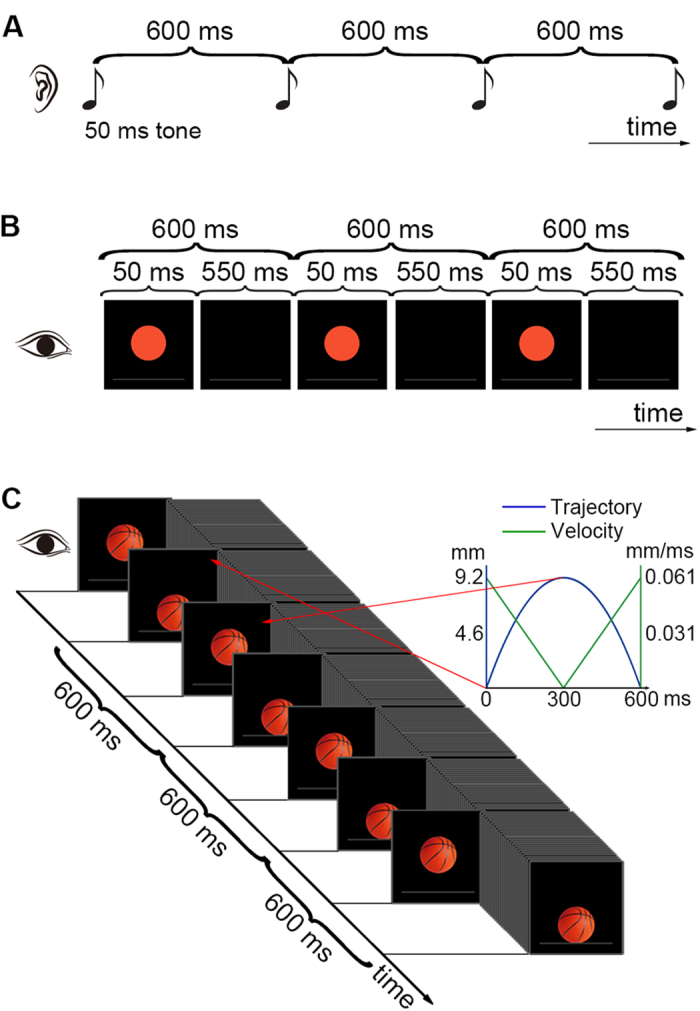
Illustration of the experimental stimuli. The subjects tapped along with an auditory tone sequence (**A**), a visual flashing ball sequence (**B**), or a visual bouncing ball sequence (**C**). Three cycles of the 600 ms IOI sequences from experiment 1 are shown. The velocity and trajectory of the visual bouncing ball are indicated in the inset of C. (The drawings in all Figures were drawn by the authors).

In experiment 1, beat synchronization was studied by having the subjects tap a finger along with a metronome^6^,^8^, which was composed of an isochronous sequence with a 600 or 900 ms IOI. There were three types of sequences (Fig. 1): the auditory tone sequence, the visual flashing ball sequence, and the visual bouncing ball sequence. The velocity of the bouncing ball was varied by simulating the effect of gravity, i.e., with a uniformly varying velocity. The acceleration of the earth’s gravity is 9.8 m/s^2^, and the movement distance of a corresponding falling object would be substantially greater than the height of a computer monitor for the typical beat intervals (300 to 900 ms). Therefore, the acceleration was 0.20 m/s^2^ for the 600 ms IOI bouncing ball sequence and was 0.09 m/s^2^ for the 900 ms IOI bouncing ball sequence (the stimuli could be viewed as representing falling objects on other possible planets with different gravitational accelerations than the earth). The assignment of the acceleration was also related to the correction of movement discontinuities. Because of the ball’s high speed at the lowest positions (see the inset of Fig. 1C), a clear movement discontinuity was observed in the preliminary testing in which the visual bouncing ball sequence was presented on a typical computer monitor using a movement-distance/ball-size ratio that was slightly larger than 1 (thus the last several steps down or first several steps up were too large). Therefore, to obtain a smooth movement, particularly at the lowest positions, a computer monitor with both a high refresh rate and a high resolution was used, and a smaller movement-distance/ball-size ratio was adopted. (See the Methods below for detailed movement parameters).

**Figure 2:**
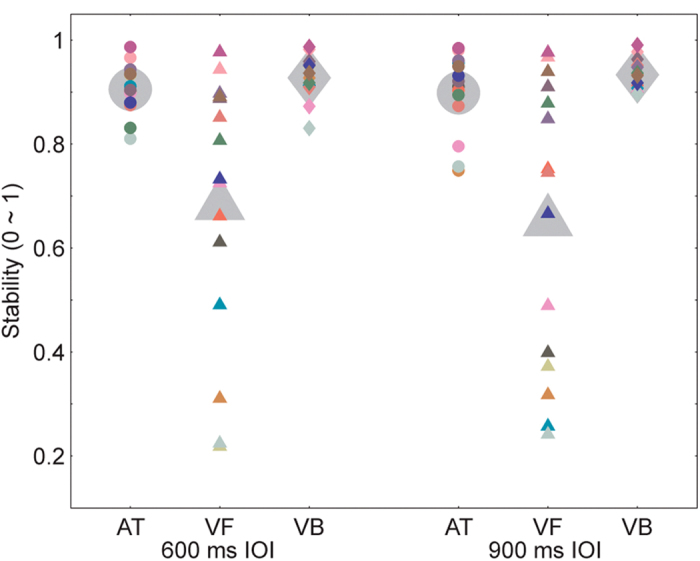
Results of experiment 1. Tapping was most stable for the visual bouncing ball sequence. The mean synchronization stabilities are indicated by the large gray marks. The data from individual subjects are indicated by the small marks of different colors, which indicate different subjects. AT, VF and VB represent the auditory tone sequence, visual flashing ball sequence, and visual bouncing ball sequence, respectively.

The stability of beat synchronization was assessed using a circular analysis method^11^,^13^. The difference between the time of a tap and the time of the corresponding event onset was measured by the relative phase (RP) on a unit circle. Synchronization stability was indexed by R, which was the length of the resultant of the RPs^11^. R ranged from 0 (randomly unstable tapping) to 1 (perfectly stable tapping) (see the Methods below for details). A two-way repeated measures analysis of variance (ANOVA) with the factors sequence type (three sequence types) and IOI type (two IOI types) showed a significant main effect for sequence type (*F*_2,28_ = 11.283, *p* = 0.001, partial η^2^ = 0.545) (Fig. 2). There was no significant main effect for IOI type and no significant interaction between the two factors. The comparisons between sequence types for individual IOIs in experiments 1 and 2 are listed in Table 1. The mean and SD of the stability (R) for all sequence types and all IOI types in all experiments are listed in Table S1. For both IOIs, tapping was more stable for the auditory tone sequence than for the visual flashing ball sequence, replicating the well-known auditory advantage of beat synchronization. However, tapping to the visual bouncing ball sequence was also more stable than tapping to the visual flashing ball sequence, and was not less stable than tapping to the auditory tone sequence (Table 1). Therefore, the auditory advantage disappeared when the realistically moving visual stimuli were adopted.

**Figure 3:**
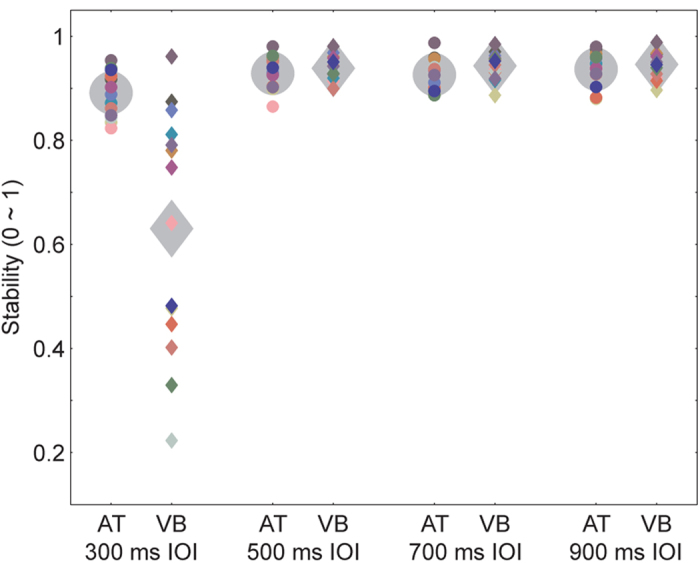
Results of experiment 2. Tapping to the visual bouncing ball sequence was less stable than tapping to the auditory tone sequence for the short 300 ms IOI, whereas the reverse pattern was exhibited for larger IOIs. The conventions are as in Fig. 2.

Since realistically moving visual stimuli were capable of improving synchronization to a visual beat, unrealistically moving visual stimuli could decrease synchronization to a visual beat. This was observed when the IOI was reduced to 300 ms (Fig. 3) in experiment 2. All subjects verbally described the 300 ms IOI visual bouncing ball sequence as “unnaturally fast”, and its associated tapping (mean stability = 0.630) was less stable than tapping to the corresponding auditory tone sequence (mean stability = 0.891) (*t*_13_ = 4.314, *p*_*corrected*_ = 0.004, η^2^ = 0.589). The poor synchronization performance for the visual bouncing ball sequence was specific to the short 300 ms IOI and was not observed for longer IOIs that ranged from 500 to 900 ms (Table 1), which replicated the above results of the 600 and 900 ms IOIs in experiment 1. Note that the poorer synchronization performance for a flash of light (e.g., the visual flashing ball sequence in the present study) than for an auditory metronome is observed for all IOIs rather than only the short 300 ms IOI^2^. A two-way ANOVA with the factors sequence type (two sequence types) and IOI type (four IOI types) showed significant interaction between the two factors (*F*_3,39_ = 19.292, *p* = 0.001, partial η^2^ = 0.597), which was consistent with the above observation that the auditory advantage was only for the 300 ms IOI, but not for the longer IOIs. In addition, the ANOVA showed significant main effects for sequence type (*F*_1,13_ = 13.924, *p* = 0.003, partial η^2^ = 0.517) and IOI type (*F*_3,39_ = 35.167, *p* < 0.001, partial η^2^ = 0.730). While the subjects reported that the 300 ms IOI bouncing ball sequence looked unnatural, the mechanism of the poor performance needs to be further determined. For example, the movement for the 300 ms IOI was less smooth than that for the longer IOIs (although the movement discontinuities at the lowest ball positions were carefully controlled for the 300 ms IOI sequence, see the Methods below), which might also contribute to the poor synchronization performance in the fastest bouncing ball condition.

**Figure 4:**
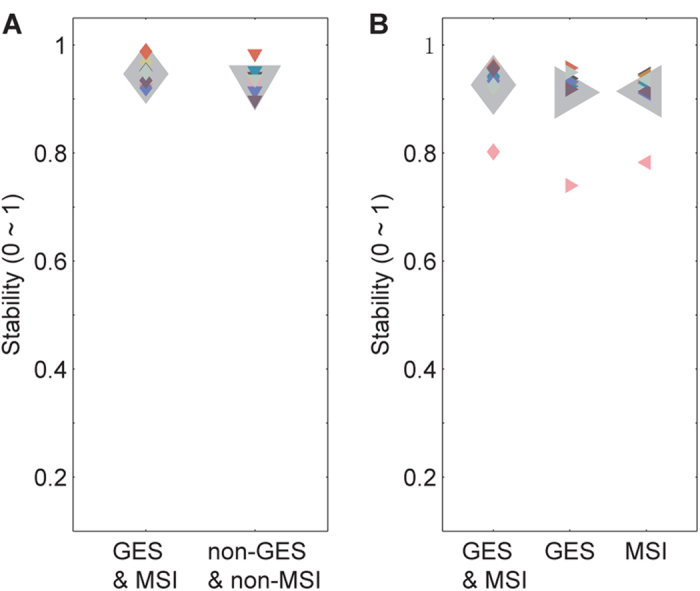
Results of control experiments 1 and 2. The bouncing ball sequence in the current study included (1) simulating the effect of gravity and (2) improving movement smoothness. The current 600 ms IOI sequence with the two manipulations was compared with a control sequence without the two manipulations in control experiment 1, and was compared with control sequences without one of the two manipulations in control experiment 2. The results of control experiment 1 (**A**) showed that combining the two manipulations significantly improved tapping stability. The results of control experiment 2 (**B**) showed that removing individual manipulations did not yield significant performance differences, although the mean stability was highest for the sequence with both manipulations (GES&MSI vs. GES: *t*_8_ = 1.679, *p* = 0.132, η^2^ = 0.^2^61; GES&MSI vs. MSI: *t*_8_ = 1.931, *p* = 0.090, η^2^ = 0.318). GES represents the gravity effect simulation and MSI represents the movement smoothness improvement. GES&MSI refers to the 600 ms IOI bouncing ball sequence as in experiment 1. Other conventions are as in Fig. 2.

Previous studies have found that the bouncing ball sequence with a velocity that varied according to a rectified sinusoid^9^,^10^ was more effective in improving synchronization than the bouncing ball sequence with a constant velocity^14^, and it has been suggested that the former sequence is more realistic than the latter sequence^10^. Notably, synchronization to the bouncing ball sequence with a velocity that varied according to a rectified sinusoid was almost as good as synchronization to an auditory metronome^9^,^10^. Inspired by these advances, we designed a bouncing ball sequence that was even more realistic by two manipulations; (1) simulating the effect of gravity and (2) improving movement smoothness. The results of experiments 1 and 2 showed that synchronization to the current bouncing ball sequence was not poorer than synchronization to the auditory tone sequence (except for the 300 ms IOI sequences). Based on the current observations, it is of interest to determine whether synchronization to the current bouncing ball sequence could be slightly better than synchronization to the bouncing ball sequence with a velocity that varied according to a rectified sinusoid^9^,^10^ (since the latter sequence has been the most effective moving stimulus in improving synchronization and its synchronization performance was slightly less stable than that observed in the auditory metronome condition). This was tested in control experiment 1, in which the current 600 ms IOI bouncing ball sequence with the two manipulations was compared with a control bouncing ball sequence without the two manipulations. We emphasize that the velocity of the control sequence was varied according to a sinusoid, rather than a rectified sinusoid; we did not replicate the stimuli in the previous studies because it has not been described in details how the rectified sinusoid was constructed^9^,^10^. Therefore, the current study did not directly compare the current bouncing ball sequence and the bouncing ball sequence with a velocity that varied according to a rectified sinusoid^9^,^10^. In addition, a typical 60 Hz refresh rate monitor was used and a large movement-distance/ball-size ratio was adopted for the control sequence. Moreover, the effect of each of the two manipulations of the current bouncing ball sequence was examined in control experiment 2, in which the current 600 ms IOI bouncing ball sequence with the two manipulations was compared with the control sequences without one of the two manipulations. The control sequence without the gravity effect simulation was the same as the current 600 ms IOI bouncing ball sequence except its velocity was varied according to a sinusoid, as described above. The control sequence without the movement smoothness improvement was the same as the current 600 ms IOI bouncing ball sequence except a typical 60 Hz refresh rate monitor was used and a large movement-distance/ball-size ratio was adopted, as described above. The results of control experiment 1 showed that combining the two manipulations (i.e., the current bouncing ball sequence (mean stability = 0.946)) significantly improved tapping stability (compared with the control sequence without the two manipulations (mean stability = 0.934)) (*t*_8_ = 2.685, *p* = 0.028, η^2^ = 0.474) (Fig. 4A). The results of control experiment 2 showed that removing individual manipulations did not yield significant performance differences (although the mean stability (see Table S1) was highest for the current bouncing ball sequence with both manipulations) (Fig. 4B).

Because the current bouncing ball sequence involved the simulation of the effect of gravity, it is reasonable that synchronization performance would degrade if the stimulus presentation was rotated 90°. This was confirmed in control experiment 3 in which the current 600 ms IOI bouncing ball sequence was rotated 90° counterclockwise. Synchronization to the non-rotated sequence (mean stability = 0.933) was more stable than synchronization to the rotated sequence (mean stability = 0.916) (*t*_8_ = 2.359, *p* = 0.046, η^2^ = 0.410). We should mention here that the results may also be influenced by the compatibility of the direction of motion of the moving stimulus and the direction of motion of the tapping finger^14^; they were compatible for the non-rotated sequence and were incompatible for the rotated sequence in control experiment 3.

In addition, with the exception of the 300 ms IOI bouncing ball sequence in experiment 2, most subjects exhibited negative relative phases of tapping (Fig. S1). This represented the negative mean asynchrony (NMA) that is typically observed in humans. We also analyzed the lag-1 autocorrelation of the inter-tap intervals (AC-1) (a negative AC-1 could suggest error correction whereas a positive or non-negative (i.e., not significantly negative) AC-1 could suggest absent or weak error correction)^9^,^14^,^15^. Consistent with previous findings^9^,^14^,^15^, negative AC-1 values were observed for the auditory tone sequence and the visual bouncing ball sequence (with the exception of the 300 ms IOI bouncing ball sequence in experiment 2) and non-negative AC-1 values were observed for the visual flashing ball sequence (Table S2). These results further supported the validity of the present data, and that the subjects were indeed synchronizing, and not simply tracking or ‘intercepting’ the moving ball stimuli.

## Discussion

The superiority of the auditory over visual modality in beat synchronization is one of the best-known results in studies of sensorimotor synchronization^3^. Recent advances, however, have shown that synchronization to a visual beat can be improved using moving stimuli instead of a conventional flashing light^9^,^10^,^14^. Specifically, synchronization to a bouncing ball was almost as good as synchronization to an auditory metronome (although a slight auditory advantage still existed), suggesting the importance of the realism of motion in improving synchronization to a visual beat^9^,^10^. By designing a bouncing ball that was even more realistic, the present study found that tapping to the bouncing ball was not less stable than tapping to an auditory metronome for the IOIs from 500 to 900 ms (with the exception of the 300 ms IOI), demonstrating that synchronization to a visual beat can indeed be as good as that to an auditory beat. Moreover, it deserves to be pointed out that, for the IOIs from 500 to 900 ms, the mean of the synchronization stability was greater for the bouncing ball than for the auditory metronome (Fig. 2, Fig. 3, and Table S1). Although the size of the effect was small and the effect did not reach significance (with Bonferroni corrections) for individual IOIs in an experiment (Table 1), the effect was reliably replicated for the IOIs longer than 300 ms in both experiments 1 and 2^16^. Therefore, the present results not only demonstrate that synchronization to a visual beat can be as good as synchronization to an auditory beat but also indicate that synchronization may be better to a visual than to an auditory beat, under optimized conditions.

The realism of moving stimuli has been suggested to be an important factor for the improvement of synchronization performance^10^. For example, the bouncing ball sequence with a velocity that varied according to a rectified sinusoid^9^,^10^ was more effective in improving synchronization than the bouncing ball sequence with a constant velocity^14^. The present results showed that if the moving stimulus was more realistic, its synchronization could be further improved and was not less stable than synchronization to an auditory beat. Given these results, however, it remains to be determined in future studies whether the realism of moving stimuli is the most critical factor in improving synchronization performance^9^. Specifically for the bouncing ball sequence in the present study, it needs to be further clarified whether the effects of the gravity effect simulation and the movement smoothness improvement could only be interpreted as the increase of stimulus realism. In addition, large subject variability has been shown in synchronization studies^9^ and therefore it is essential to examine the current results in future studies with different samples of subjects.

The current finding has important implications for the neural and evolutionary substrates of beat synchronization. Tighter connections between the auditory and motor cortices than between the visual and motor cortices have been proposed for sensorimotor synchronization^4^,^5^,^6^. The simple tapping task employed in the present study recruits a striato-thalamo-cortical loop, in which the basal ganglia (particularly the putamen) plays a crucial role in coordinating the input timing information in the auditory or visual areas and the action timing in the motor areas ^for review, see^ 8. Using a visual beat consisting of moving stimuli, a recent study showed that basal ganglia activation was associated with synchronization stability rather than modality specificity^11^, which is consistent with the current behavioral evidence that does not support the modality bias of beat synchronization. A vocal learning and rhythmic synchronization (VLRS) hypothesis proposed that vocal learning drives the evolution of beat synchronization and that beat synchronization is a brain function that is shared by humans and only a few other species with vocal learning ability^6^. The VLRS hypothesis is based upon the assumption that vocal learning and beat synchronization are supported by general tighter connections between the auditory and motor cortices than between the visual and motor cortices^6^, whereas this assumption is not supported by the current finding, as discussed above. It should be noted that the bouncing ball sequence as used in the current study presented the perceptual system with continuous information (motion), while the auditory tone sequence used discretely-timed information. Therefore, the current results may suggest a useful refinement to the VLRS hypothesis that the general-tighter-connection assumption may only apply to circuits involved in the timing of discretely-timed periodic events.

In summary, the present study found that synchronization to a bouncing ball with a realistic motion trajectory was not less stable than synchronization to an auditory metronome. This finding challenges the auditory advantage of beat synchronization and calls for a reconsideration of the biological substrates of beat synchronization that were proposed based on the modality bias.

## Methods

### Participants

Fifteen subjects (all right-handed, four males, mean age ± SD 22.7 ± 2.9 years), fifteen subjects (all right-handed, four males, mean age ± SD 22.6 ± 1.6 years), nine subjects (all right-handed, two males, mean age ± SD 24.3 ± 4.4 years), nine subjects (all right-handed, three males, mean age ± SD 23.4 ± 4.0 years), and nine subjects (all right-handed, four males, mean age ± SD 23.1 ± 1.9 years) participated in experiment 1, experiment 2, control experiment 1, control experiment 2, and control experiment 3, respectively. Three subjects in experiment 1 (playing piano for five, ten, and five years, respectively), three subjects in experiment 2 (playing piano for ten, five, and seven years, respectively), one subject in control experiment 1 (playing piano for ten years), and two subjects in experiment 3 (both playing piano for five years) reported musical experience. Two subjects participated in experiments 1 and 2; one subject participated in experiment 1, experiment 2, and control experiment 3; one subject participated in experiment 1, experiment 2, control experiment 2, and control experiment 3; and one subject participated in experiment 1, control experiment 2, and control experiment 3. All subjects had normal hearing and had normal or corrected-to-normal vision. The research protocols in this study were approved by the Institutional Review Board of Psychology Department of Sun Yat-Sen University. All subjects gave written informed consent. A typical tapping task involves approximately nine subjects^17^, which was the number of subjects in the present control experiments. To further validate and confirm the new finding of the present study, fifteen subjects were recruited in experiments 1 and 2. One subject in experiment 2 was excluded from analyses because the subject reported difficulty in performing the task and did not finish the experiment. The methods were carried out in accordance with the approved guidelines.

### Stimuli and procedure

The subjects sat in front of an LCD computer monitor (120 Hz refresh rate, 1920 × 1080 resolution, and 53.1 cm × 29.8 cm) with a viewing distance of 60 cm and wore a headset. In all the experiments, the subjects were asked to tap in synchrony with isochronous sequences using the index finger of their preferred hand on a key of a computer keyboard. The keyboard used in the present study was a standard Dell computer keyboard, which introduced a systematic latency (about 10 ms) in checking the tapping. As a result, the effective temporal resolution of the keyboard was about ± 10 ms.

In experiment 1, three types of isochronous sequences with either a 600 or a 900 ms inter-onset interval (IOI) were presented: the auditory tone sequence, the visual flashing ball sequence, and the visual bouncing ball sequence (Fig. 1). For the auditory tone sequence, a pure tone (600 Hz, 50 ms duration) was presented every 600 ms or 900 ms for the 600 and 900 ms IOI sequences, respectively. An orange ball was displayed at the center of the computer screen on a black background. The ball was 1.74 cm in diameter. A 3.54 × 0.06 cm white bar was 0.92 cm below the bottom edge of the ball. The subjects were required to fixate on the ball and to maintain attention on the auditory task (so that the subjects would not look around and be attracted by other factors). For the visual flashing ball sequence, the ball flashed every 600 ms or 900 ms for the 600 and 900 ms IOI sequences, respectively (the ball lasted for 50 ms and disappeared for the remaining IOI time). For the visual bouncing ball sequence, the ball was replaced with a realistic orange basketball. The basketball continually moved 0.92 cm (movement distance) down and touched the bar, and then moved up to the initial position. The velocity of the bouncing ball was varied by simulating the effect of gravity, i.e., with a uniformly varying velocity. The acceleration was 0.20 m/s^2^ for the 600 ms IOI bouncing ball sequence and was 0.09 m/s^2^ for the 900 ms IOI bouncing ball sequence. The ball size, the movement distance, and the movement-distance/ball-size ratio of 0.529 were kept constant across different IOIs. Each movement step lasted for a frame. The stimuli were presented using Psychtoolbox (http://psychtoolbox.org). Event onsets referred to the onsets of the auditory tone, the onsets of the visual flashes, or the moment when the ball touched the bar for the three sequence types, respectively. Stimulus presentation was self-paced (the subjects pressed the space bar to start a sequence). Each sequence had 55 events (54 IOIs or circles). A 2 s blank screen was presented at the beginning and end of the auditory tone or visual flashing ball sequence, and a 2s-IOI/2 blank screen was presented at the beginning and end of the visual bouncing ball sequence (the ball moved down from the center of the screen at the beginning of a sequence and moved back to the screen center at the end; the event onset referred to the ball touching the bar at the lowest position). (See Video S1 for the demo of the 600 ms IOI visual bouncing ball sequence). Each sequence type was repeated six times. The orders of the IOI types and the sequence types were counterbalanced across the subjects.

Experiment 2 was the same as experiment 1 except 1) there were four IOI types (300, 500, 700, and 900 ms) and two sequence types (auditory tone and visual bouncing basketball sequences); and 2) the movement distance of the ball was 0.77 cm and the movement-distance/ball-size ratio was 0.443 (this ratio was smaller than that in experiment 1. This was done to avoid movement discontinuities at the lowest ball positions for the 300 ms IOI sequence). The accelerations were 0.68 m/s^2^, 0.25 m/s^2^, 0.13 m/s^2^, and 0.08 m/s^2^ for the 300, 500, 700, and 900 IOIs, respectively.

In control experiment 1, using a 600 ms IOI, the current visual bouncing ball sequence was compared with a control visual bouncing ball sequence. For the control sequence, a typical LCD monitor (60 Hz refresh rate, 1440 × 900 resolution, and 41 cm × 25 cm) was used. The movement distance is 1.74 cm and the movement-distance/ball-size ratio was 1. The velocity of the ball was calculated according to a sinusoid. Other experimental settings were as in experiment 1.

In control experiment 2, the current 600 ms IOI visual bouncing ball sequence was compared with two control visual bouncing ball sequences. The control sequences were the same as the current bouncing ball sequence with the exception that in one the velocity of the ball was calculated according to a sinusoid (Fig. S2) and in the other a typical LCD monitor was used and the movement-distance/ball-size ratio was 1 (as manipulated in control experiment 1). Other experimental settings were as in experiment 1.

In control experiment 3, the current 600 ms IOI visual bouncing ball sequence was compared with a control sequence that was produced by rotating the presentation of the current 600 ms IOI visual bouncing ball sequence counterclockwise by 90°. Other experimental settings were as in experiment 1.

### Data analyses

We used circular analysis methods because they are more suitable for the variable periodic synchronization data than the standard linear analysis methods^7^,^11^,^13^,^14^. The analyses were performed using the CircStat toolbox^18^ programmed with MATLAB (The Mathworks, Natick, MA, USA). The difference between the time of a tap and the time of the corresponding event onset (asynchrony) was measured by the relative phase (RP) on a unit circle (-pi to pi. 0 indicates perfect alignment between taps and events; negative and positive values indicate taps preceding or following events, respectively; and ± pi indicates taps midway between events). Synchronization stability was indexed by R, which was the length of the resultant (i.e., average of vectors) of the RPs and was calculated by abs(sum(exp(i*RP))/n) (n indicates the number of the RPs)^11^. R ranged from 0 (unstable tapping with uniformly distributed relative phases) to 1 (perfectly stable tapping with a unimodal distribution of relative phases). Correspondingly, mean asynchrony was indexed by the angle of the resultant of the RPs and was calculated by angle(sum(exp(i*RP))/n). For the illustration of the circular histogram of the RPs (see Fig. S1F), each bin encompassed a radian range of 1/10 pi (0–1/10 pi, 1/10 –1/5 pi, etc.), with 20 bins in a circular distribution. The radial axis was set between 0–1, which represented the proportion to the total number of taps. The resultant of the RPs was represented by the red arrow, with its length indicating R and its angle indicating mean asynchrony. The taps to the first five events in a sequence were omitted from the analyses because synchronization typically requires a few taps to stabilize. In addition, the relation between adjacent inter-tap intervals (ITI) was assessed by lag-1 autocorrelation of the ITIs (AC-1)^9^,^11^. Greenhouse-Geisser corrections were applied to all ANOVA analyses. Bonferroni corrections were applied to all t-tests and corrected *p* values ≤ 0.05 were considered significant. All t-tests were two-tailed.

## Additional Information

**How to cite this article**: Gan, L. *et al.* Synchronization to a bouncing ball with a realistic motion trajectory. *Sci. Rep.* **5**, 11974; doi: 10.1038/srep11974 (2015).

## Supplementary Material

Supplementary Video S1

Supplementary Video S2

Supplementary Information

## Figures and Tables

**Table 1:**
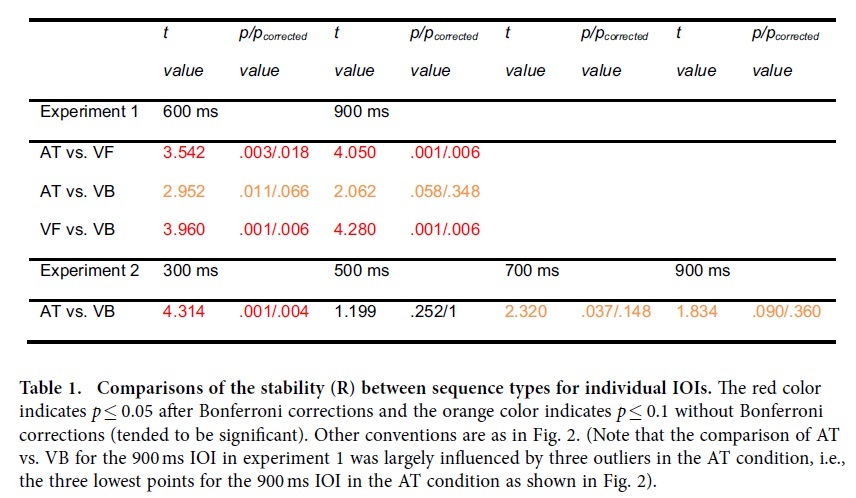
Comparisons of the stability (R) between sequence types for individual IOIs.
