# Supplementary material for: Synchronization to a bouncing ball with a realistic motion trajectory
Source: Sci Rep. 2015 Jul 7;5:11974. doi: 10.1038/srep11974 (PMC4493690; doi:10.1038/srep11974)
Supplement: Supplementary Information [file srep11974-s3.pdf]

Title: Synchronization to a bouncing ball with a realistic motion trajectory

Authors: Lingyu Gan, Yingyu Huang, Liang Zhou, Cheng Qian, Xiang Wu*

Supplementary Information

Figure S1 to S2; Table S1 to S2; Videos S1 to S2.

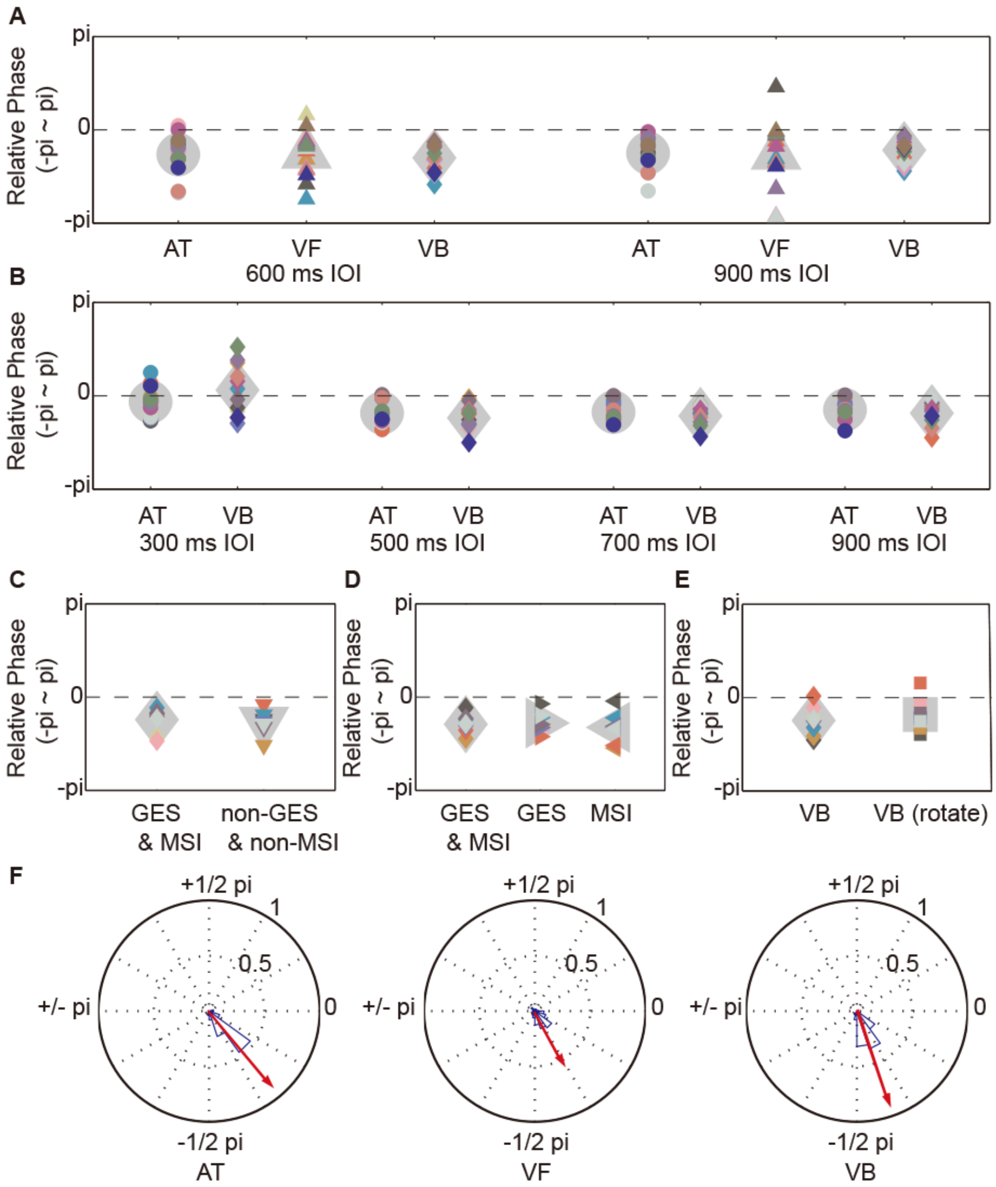

Fig. S1. Illustration of the negative mean asynchrony (NMA). With the exception of the 300 ms IOI bouncing ball sequence in experiment 2, most subjects exhibited negative relative phases (RP) of tapping, representing the NMA that is typically observed in humans. The data of experiment 1, experiment 2, control experiment 1, control experiment 2, and control experiment 3 are shown in A, B, C, D, and E respectively. The example (one trial for each sequence type) angular distributions of the tapping phases relative to the beat with a 600 ms IOI from one representative subject in experiment 1 are illustrated in F on a unit circle. The radial axis is set between 0–1, which represents the proportion to the total number of taps. The resultant of the RPs is represented by the red arrow, with its length indicating stability (R) and its angle indicating mean asynchrony. Other conventions are as in Fig. 2 and Fig. 4.

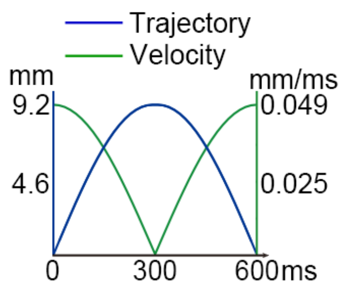

Fig. S2. Illustration of the velocity and trajectory of the control visual bouncing ball with a sinusoidally varying velocity in control experiment 2. This control 600 ms IOI bouncing ball (corresponding to "MSI" in Fig. 4B) was the same as the 600 ms IOI bouncing ball in experiment 1, with the exception that its velocity was varied according to a sinusoid. (Note that the control bouncing ball in control experiment 1 also had a sinusoidally varying velocity, but with a long (1.74 cm) movement distance). Conventions are as in Fig. 1.

	Mean	*SD*	*Mean*	*SD*	*Mean*	*SD*	*Mean*	*SD*
Experiment 1	600 ms		900 ms					
AT	.905	.046	.898	.075				
VF	.682	.259	.651	.276				
VB	.927	.040	.933	.029				
Experiment 2	300 ms		500 ms		700 ms		900 ms	
AT	.891	.044	.929	.032	.926	.030	.937	.032
VB	.630	.233	.939	.023	.943	.025	.946	.024
Control experiment 1	600 ms							
GES&MSI (VB)	.946	.022						
non-GES&non-MSI	.934	.025						
Control experiment 2	600 ms							
GES&MSI (VB)	.926	.049						
GES	.912	.066						
MSI	.914	.051						
Control experiment 3	600 ms							
VB	.933	.019						
VB (rotate)	.916	.032						

Table S1. The mean and SD of the stability (R) for all sequence types and all IOI types in all experiments. Conventions are as in Fig. 2 and Fig. 4. Note that in control experiments 1 and 2 GES&MSI refers to the 600 ms IOI bouncing ball sequence as in experiment 1.

	Mean	*SD*	*Mean*	*SD*	*Mean*	*SD*	*Mean*	*SD*
Experiment 1	600 ms		900 ms					
AT	-.201	.085	-.266	.113				
VF	-.006	.164	-.003	.193				
VB	-.275	.101	-.300	.101				
Experiment 2	300 ms		500 ms		700 ms		900 ms	
AT	-.259	.116	-.241	.092	-.258	.092	-.316	.129
VB	-.060	.199	-.325	.105	-.297.	.098	-.336	.089
Control experiment 1	600 ms							
GES&MSI (VB)	-.279	.095						
non-GES&non-MSI	-.336	.083						
Control experiment 2	600 ms							
GES&MSI (VB)	-.295	.115						
GES	-.331	.121						
MSI	-.299	.123						
Control experiment 3	600 ms							
VB	-.221	.107						
VB (rotate)	-.249	.109						

Table S2. The mean and SD of lag-1 autocorrelation (AC-1) for all sequence types and all IOI types in all experiments. Conventions are as in Table S1.

Video S1. The video demo of the 600 ms IOI visual bouncing ball sequence in experiment 1. Four cycles (5 beats) are shown.

Video S2. The video demo of the control bouncing ball sequence in control experiment 1. Four cycles (5 beats) are shown.
